# Quantitative analysis of the CD4^+^ T cell response to therapeutic antibodies in healthy donors using a novel T cell:PBMC assay

**DOI:** 10.1371/journal.pone.0178544

**Published:** 2017-05-31

**Authors:** Heidi S. Schultz, Stine Louise Reedtz-Runge, B. Thomas Bäckström, Kasper Lamberth, Christian R. Pedersen, Anne M. Kvarnhammar, on behalf of the ABIRISK consortium

**Affiliations:** 1 Immunogenicity Prediction and Tolerance, Global Research, Novo Nordisk A/S, Måløv, Denmark; 2 NCD Project Management, Global Research, Novo Nordisk A/S, Måløv, Denmark; 3 Immunogenicity Assessment, Global Research, Novo Nordisk A/S, Måløv, Denmark; Fujita Health University, School of Medicine., JAPAN

## Abstract

Many biopharmaceuticals (BPs) are known to be immunogenic in the clinic, which can result in modified pharmacokinetics, reduced efficacy, allergic reactions and anaphylaxis. During recent years, several technologies to predict immunogenicity have been introduced, but the predictive value is still considered low. Thus, there is an unmet medical need for optimization of such technologies. The generation of T cell dependent high affinity anti-drug antibodies plays a key role in clinical immunogenicity. This study aimed at developing and evaluating a novel *in vitro* T cell:PBMC assay for prediction of the immunogenicity potential of BPs. To this end, we assessed the ability of infliximab (anti-TNF-α), rituximab (anti-CD20), adalimumab (anti-TNF-α) and natalizumab (anti-α4-integrin), all showing immunogenicity in the clinic, to induce a CD4^+^ T cells response. Keyhole limpet hemocyanin (KLH) and cytomegalovirus pp65 protein (CMV) were included as neo-antigen and recall antigen positive controls, respectively. By analyzing 26 healthy donors having HLA-DRB1 alleles matching the European population, we calculated the frequency of responding donors, the magnitude of the response, and the frequency of BP-specific T cells, as measured by ^3^[H]-thymidine incorporation and ELISpot IL-2 secretion. KLH and CMV demonstrated a strong T cell response in all the donors analyzed. The frequency of responding donors to the BPs was 4% for infliximab, 8% for adalimumab, 19% for rituximab and 27% for natalizumab, which is compared to and discussed with their respective observed clinical immunogenicity. This study further complements predictive immunogenicity testing by quantifying the *in vitro* CD4^+^ T cell responses to different BPs. Even though the data generated using this modified method does not directly translate to the clinical situation, a high sensitivity and immunogenic potential of most BPs is demonstrated.

## Introduction

Biopharmaceuticals (BPs), such as monoclonal antibodies (mAbs) are widely used for the treatment of autoimmune disease, and cancer. A major concern regarding treatment with therapeutic proteins is the risk of provoking an unwanted immune response, such as the development of anti-drug antibodies (ADAs). ADAs can potentially decrease the efficacy of the BPs, modify clearance, induce hypersensitivity reactions or cause severe adverse events [1, 2]. Many factors contribute to the immunogenicity of BPs, including product-, disease-, treatment- and patient-related factors [3]. Product-related factors include intrinsic factors like homology to human amino acids sequences and posttranslational modifications, and extrinsic factors such as dose, formulation, route and frequency of administration, aggregates and impurities [4]. For the patient, elements like genetic factors including HLA type, gender and concomitant medication are contributing elements [5]. Regardless of how immunogenicity is triggered, it is evident that the formation of high affinity Abs to BPs is CD4^+^ T cell dependent [5, 6]. A T cell dependent Ab response relies on T cell recognition of protein-derived epitopes that have been taken up, processed and displayed by HLA class II on antigen presenting cells (APCs). Because of polymorphisms in the HLA class II genes, the CD4^+^ T cell epitopes can differ between individuals. [7]. The importance of a potent T cell epitope has been described in several studies [8–11]. In fact, amelioration of immunogenicity has been observed by removing T cell epitopes from e.g. IFNβ1b [12] and mAbs [13]. Consequently, detection of BP-specific T cells in healthy naive donors is considered as one of the major approaches to assess immunogenicity risk. Several methods to evaluate T cell responses have been published and applied during drug development to reduce the risk for immunogenicity in the clinic. These include peripheral blood mononuclear cell (PBMC)-based assays [14], dendritic cell (DC):T cell assays [15, 16] and more complex assays where naïve T cells are amplified polyclonally [17] or antigen-specifically [18, 19].

Numerous biological products have been approved by FDA. When reviewing the label of these compounds, immunogenicity has been reported in 89% of the cases wherein half of these incidences impacts the efficacy of the drug [20]. One of the most important and diverse therapeutic classes of BPs in the clinic are the therapeutic mAbs. Examples of mAbs with exhaustive documented clinical immunogenicity are the anti-TNF-α mAbs infliximab (Remicade^®^) and adalimumab (Humira^®^), as well as the anti-α4-integrin mAb natalizumab (Tysabri^®^). They are all used in treatment of inflammatory disease and have been observed to have high incidences (up to 87%) of ADA formation [21–23]. Rituximab, an anti-CD20 mAb used for treatment of lymphoma and inflammatory diseases, shows high incidences of ADA in the latter [24, 25].

Due to the safety issues associated with immunogenicity, it is of great importance to reduce the risk for immunogenicity in the clinic. Currently, no pre-clinical immunogenicity tools can predict clinical immunogenicity. Nevertheless, in this study we are trying to address the relation between an *in vitro* T cell assay and clinical immunogenicity. As a part of managing these unwanted immunogenicity associated risks, an immense effort has been made by the ABIRISK consortium (www.abirisk.eu) of the European Innovative Medicines Initiative. The major goals of the consortium are to improve methods for immunogenicity prediction and ADA assessment, as well as to establish common definitions and terms related to immunogenicity [26].

Still acknowledging the caveats and limitations of immunogenicity prediction, the purpose of the present study was to develop a high throughput and sensitive method to evaluate the CD4^+^ T cell response of healthy donors to specific BPs such as neo-antigens, like therapeutic mAbs. Based on the commonly used CD8^+^ T cell-depleted PBMC and DC:T cell assays we developed a novel *in vitro* hybrid T cell assay that uses purified CD4^+^ T cells co-cultured with irradiated PBMCs. By evaluating a cohort of 26 healthy donors for their responses to KLH, CMV, infliximab, rituximab, adalimumab and natalizumab, in terms of proliferation and IL-2 secretion, we determined the frequency of responding donors, the magnitude of the response, as well as the BP-specific T cell repertoire. Our novel T cell:PBMC assay demonstrated a higher sensitivity compared to the standard CD8^+^ T cell-depleted PBMC assay and showed a high *in vitro* immunogenicity potential for most of the BPs evaluated.

## Materials and methods

### Proteins

The primary antigen KLH (Thermo Fisher Scientific, Rockford, IL, USA) was prepared as followed; KLH was dissolved in ultrapure water, left on ice for 30 minutes and dialyzed (Thermo scientific, Rockford, IL, USA) against PBS followed by a 30 minutes centrifugation (3220 x *g*) to remove undissolved particles. The final assay concentration for KLH was 30 μg/ml. Protective antigen (PA) from *Bacillus anthracis* (List Biological Labs, Campbell, CA, USA) was used in the assay at a final concentration of 3 μg/ml. Cytomegalovirus pp65 protein (CMV; Miltenyi Biotec, Lund; Sweden) was used in the assay at 2 μl/ml (concentration unknown). Infliximab (10 mg/ml), rituximab (10 mg/ml), adalimumab (50 mg/ml) and natalizumab (20 mg/ml) were obtained from the ABIRISK consortium (Novartis, Basel, Switzerland). The BPs were aliqouted and stored at -80°C according to the instructions provided. The Abs were used in the assay at 45 μg/ml (0.3μM).

### T cell assay setup

Blood donations were obtained from screened healthy volunteers via the Danish Blood Bank under informed consent, according to the protocol H-D-2008-113 for research use approved by the Danish Scientific Ethical Committee Region Hovedstaden (Legislative Order No. 402 of May 28^th^, 2008). Donations were fully anonymous to Novo Nordisk A/S employees. PBMCs were purified by Ficoll-Plaque Plus (GE Healthcare, Uppsala, Sweden) density centrifugation. Red blood cells were lysed using RBC lysis buffer (eBioscience, San Diego, CA, USA) and the PBMCs washed twice in PBS. A fraction of the PBMCs was γ-irradiated at 3000 rads to prevent cell division to ensure that the responses seen solely are CD4^+^ T cell-mediated. From the remaining fraction, CD4^+^ T cells were isolated using a CD4^+^ T cell enrichment kit (Easysep, Stemcell Technologies, Grenoble, France). CD4^+^ T cell purity was assessed by flow cytometry and was within the range of 93.0±4.8%. The CD4^+^ T cells were co-cultured at 37°C in 5% CO_2_ in serum-free Optimizer medium (Gibco, Grand Island, NY, USA) supplemented with Optimizer T-cell expansion supplement, 2 mM GlutaMAX (Gibco, Grand Island, NY, USA), 50 U/ml Penicillin and 50 μg/ml Streptomycin (Gibco, Grand Island, NY, USA) at a ratio 1:2 with the autologous irradiated PBMCs. After six-eight days, proliferation and IL-2 secretion were determined.

### T cell proliferation

To assess T cell proliferation, 1x10^5^ CD4^+^ T cells were co-cultured with 2x10^5^ autologous PBMCs in 96-well plates in the absence or presence of BPs and control Ags. Cells were cultured for five or seven days before being pulsed with 0.5 μCi ^3^[H]-thymidine (Perkin Elmer, Groningen; Netherlands) for 18 hours. The cells were harvested using a 96-well cell FilterMate harvester (PerkinElmer, Warrenville road IL, USA). ^3^[H]-thymidine incorporation was measured by liquid scintillation counting using a TopCount NXT (Perkin Elmer, Warrenville road IL, USA). Data was analyzed using GraphPad Prism 6.0 software (Graphpad Software version 6, La Jolla, CA, USA). Each sample was tested in sextuplicates.

### IL-2 ELISpot assay

For ELISpot analysis, 5x10^5^ CD4^+^ T cells were co-cultured with 1x10^6^ autologous PBMCs in 24-well plates in the absence or presence of BPs and control antigens for six days. To all the conditions, 5 μl/ml anti-CD28/CD49d mAbs (BD Biosciences, San Jose, CA, USA) co-stimulatory reagent was added. Pre-coated IL-2 ELISpot (Mabtech, Nacka Strand, Sweden) plates were washed in PBS prior to conditioning the plate with Optimizer medium with 10% FBS (Gibco, Grand Island, NY, USA) for 30 minutes. The cultured cells were washed twice, plated on the ELISpot plate in triplicates and re-stimulated with the BPs. After 18 hours the ELISpot plate was developed according to manufacturer’s instructions. Plates were scanned on an ImmunoSpot^®^ S5 analyzer and the total number of spots per well (spw) was determined using ImmunoSpot^®^ 5.0.9 analyzer software (CLT, Inc., Shaker Heights, OH, USA).

### HLA genotyping

PBMCs (1-2x10^6^) were snap frozen on dry ice and stored at -80°C until analysis. The PBMC samples were shipped to ProImmune (Oxford, UK) for HLA class II typing. HLA genotyping was performed using PCR-sequence specific oligonucleotides (PCR-SSOP) to resolve major allele groups to 4 digits.

### T cell assay analysis

Positive responses to the compounds were based on both statistical and empirical thresholds. For the statistical threshold a positive hit was found when p<0.05 comparing counts per minute (cpm) for T cell proliferation (n = 6) or spw for ELISpot (n = 3) of BP treated wells against baseline wells, using a two-tailed unpaired student’s t-test (GraphPad Prism version 6, La Jolla, CA, USA). The empirical threshold was based on a stimulation index (SI)>2, where SI was calculated from cpm_Ag_/cpm_baseline_ or spw_Ag_/spw_baseline_. For a given compound, a positive response was defined to have SI>2 and p<0.05. The distribution of rare sets of T cell precursors has been shown to follow a Poisson distribution [27, 28]. The frequency of BP-specific CD4^+^ T cells was calculated using the following formula: Frequency = -ln(negative wells/total well tested)/(CD4^+^ T cells/well) [19]. Wells were scored positive when they exceeded the value > 2 x average cmp_baseline_. When all wells were positive for a T cell response we set the number of negative wells to 0.1, since the formula cannot accept the value 0.

## Results

### Development of a novel T cell:PBMC assay

**Fig 1:**
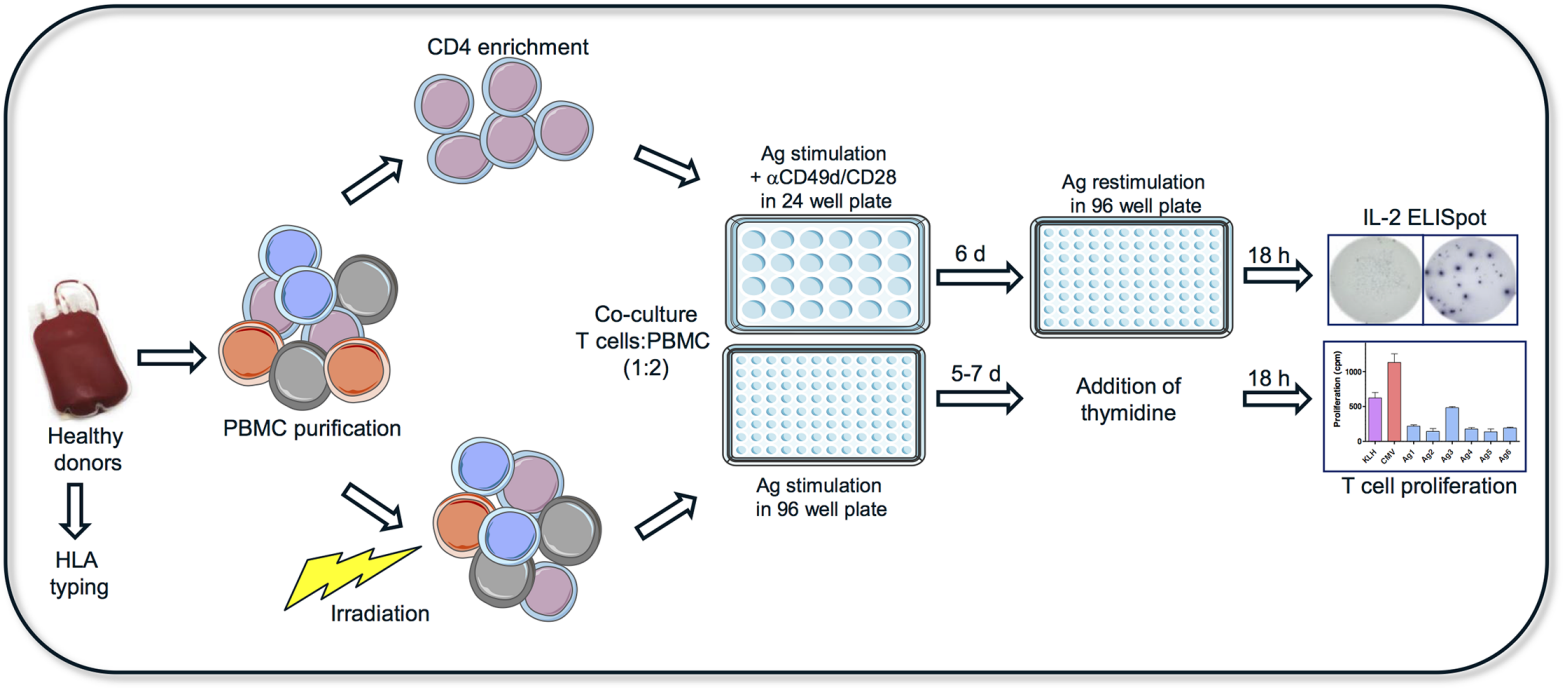
A novel T cell:PBMC assay to detect CD4^+^ T cell response to BPs in healthy donors. The figure shows a schematic representation of the T cell:PBMC assay format: PBMCs were obtained from naive healthy donors. CD4^+^ T cells were enriched and plated in 24-well or 96-well plates at respectively 5x10^5^ or 1x10^5^ cells/well in multiple wells containing irradiated allogeneic PBMC at a concentration of either 2x10^5^ or 1x10^6^ cells/well. The co-cultured cells were challenged with KLH (30 μg/ml), CMV (2 μl/ml), infliximab, rituximab, adalimumab and natalizumab (all 45 μg/ml). To the 24-wells for ELISpot analysis 5 μl/ml anti-CD28/CD49d mAbs were added. At day six, the 24-well cell cultures were washed thoroughly, re-stimulated with the corresponding compounds and plated in triplicates in 96-well plates for 18 hours prior to spot detection. At day six and eight, proliferation (n = 6) was measured after 18-h pulse with ^3^[H]-thymidine. Reprint from Servier Medical Art by Servier under a CC BY license with permission from Servier Medical Art, original copyright Creative Commons Attribution 3.0 Unported License.

To develop a sensitive and high throughput T cell assay we combined and optimized the current assays used by contract research organizations (CRO), including PBMC and DC:T cell assays from Antitope Ltd (EpiScreen^TM^), Lonza (EpiBase^TM^), ProImmune^Ltd^ (REVEAL^®^), EpiVax Inc. and ImmunXperts. The parameters considered were media, cell culture conditions, cell number ratio, antigen-presenting cells (APCs), culture time, bulk culture vs single well, co-stimulation, concentration, number of BP stimulations, readouts and analyses. The optimization of the assay culture conditions was achieved using well-known immunogenic proteins; the primary antigens KLH and PA, and the recall antigen CMV. The optimized method is shown in a schematic overview in Fig 1. The main novelty with the assay is the combination and ratio of purified CD4^+^ T cells and the use of irradiated PBMCs as APCs.

**Fig 2:**
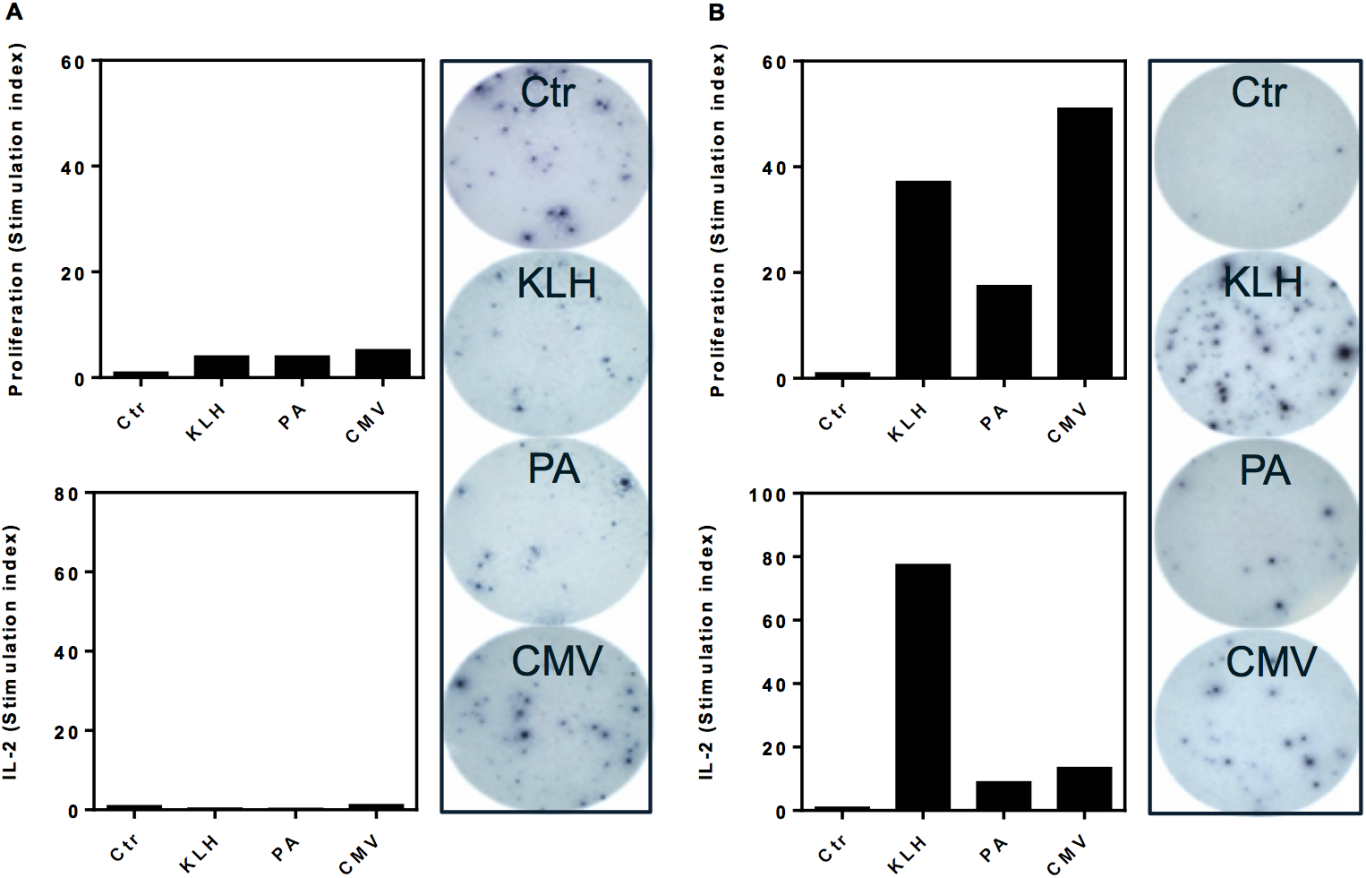
Responses to KLH, PA and CMV. PBMCs from a healthy donor were isolated and used for both (A) CD8^+^ T cell-depleted PBMC assay and (B) our optimized T cell:PBMC assay. The cultures were challenged with keyhole limpet hemocyanin (KLH, 30 μg/ml), Protective antigen (PA, 3 μg/ml) and cytomegalovirus (CMV, 2μl/ml). Proliferation was measured by ^3^[H]-thymidine incorporation at day six and IL-2 secretion was measured by ELISpot analysis at day seven. The proliferative response in counts per minute (cpm) and ELISpot IL-2 secretion (spw) was converted to stimulation index (SI). Shown are graphs of SI of proliferation and IL-2 secretion, which is also visualized by pictures. Shown is one representative donor out of four.

Comparing the optimized T cell:PBMC assay with a standard CD8^+^ T cell-depleted PBMC assay, an increased response to KLH, CMV and PA for both IL-2 secretion and proliferation was observed (Fig 2). For the CD8^+^ T cell-depleted PBMC assay, the SI values for the antigens were observed to be between 0.4–1.3 for IL-2 secretion and 4–5 for proliferation. Compared to these, the SI values for the T cell:PBMC assay were approximately 10–40 fold higher with SI values between 9–57 for IL-2 secretion and 17–51 for proliferation. By reducing the background, the T cell:PBMC assay was found to have a higher sensitivity to the model antigens compared to CD8^+^ T cell-depleted PBMC assay.

### Assessment of T cell response to biopharmaceuticals

**Fig 3:**
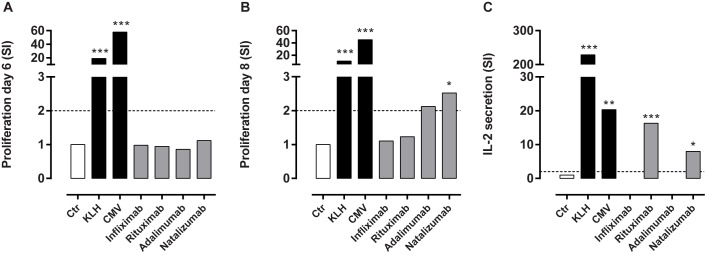
Example of a donor. One representative donor’s response to KLH (30 μg/ml), CMV (2 μl/ml), infliximab, rituximab, adalimumab and natalizumab (all 45 μg/ml) was assessed in the T cell:PBMC assay. The ability to elicit an Ag-specific response was detected by proliferation and IL-2 ELISpot. The response to untreated is included as a negative control. Proliferation was measured by ^3^[H]-thymidine incorporation in sextuplet at (A) day six and (B) day eight, shown as stimulation index. IL-2-producing T cells were identified by ELISpot analysis as shown by (C) stimulation index and as (D) visualized by pictures. The statistical difference indicated is based on raw data; cpm for T cell proliferation (n = 6) or spw for ELISpot (n = 3) using an unpaired student t test.

By applying our optimized assay, cells from a cohort of 26 healthy volunteers were exposed to the four mAbs (listed in Table 1). Each donor was assessed by determining their individual responses to the controls and BPs, respectively. A representative example of a donor is given in Fig 3. This donor was observed to respond positively to KLH, CMV and natalizumab by means of proliferation, and to KLH, CMV, rituximab and natalizumab in terms of IL-2 secretion. Hence, based on the summary of positive responses in these two assays, this donor was stated to respond positively to KLH, CMV, rituximab and natalizumab.

**Table 1:** Summary of research data and characteristics of BPs from European clinical trials and studies.

			*In vitro* response (present study)		Clinical and study data		
BP name	Route of injection	Target	Frequency of responding donors (%)	Per 10^6^ cells	Indication	ADA response (%)	References
Infliximab (chimeric)	IV	TNF-α	3.8	0.2	RA	13–44	[29–31]
					Crohn's disease	61	[32]
					Cutaneous systemic sclerosis	31	[33]
					AS	29	[34]
Rituximab (chimeric)	IV	CD20	19.2	0.6	RA	9	[35]
					SLE	36	[36]
					Primary Sjogren's syndrome	27	[37]
					Vasculitis	25	[36]
					Severe pemphigus	18	[38]
Adalimumab (human)	SC	TNF-α	7.7	0.4	RA	2–87	[31, 39, 40]
					AS	22.4	[41]
Natalizumab (humanized)	IV	α4-integrin	26.9	2.2	MS	4–5	[23, 42]

RA: Rheumatoid arthritis; AS: Ankylosing spondylitis; MS: Multiple sclerosis; SLE: Systemic lupus erythematosus; IV: intravenous injection; SC: subcutaneous injection

### Magnitude of T cell response to biopharmaceuticals

**Fig 4:**
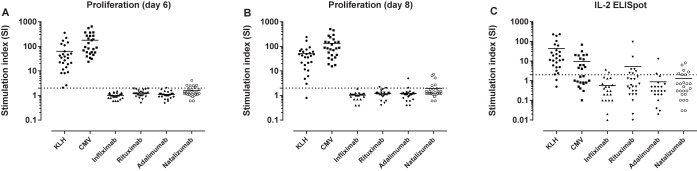
Mean magnitude of the BPs in the test population. Scatter plot summarizing the mean stimulation index value of all the donors in the study population. Shown is (A) proliferation day six, (B) proliferation day eight and (C) IL-2 secretion. The dotted line indicates SI = 2, which is the cut-off value for a positive response.

The abovementioned way of calculating responding donors reduces the data into a positive/negative distribution and does not consider the strength of the response. Therefore, the mean SI value of the entire study population was also examined. Fig 4 shows the magnitude for the individual donors for each test compound for proliferation and IL-2 secretion, respectively. Of the tested compounds, the positive controls, KLH and CMV, had the highest mean SI responses, ranging between 45–65 and 10–180, respectively. Of the BPs, infliximab, rituximab, adalimumab and natalizumab had a correspondingly mean SI value of 1.0, 1.2, 1.2 and 1.8 in average for proliferation (Fig 4A and 4B). For IL-2 secretion the mean SI value was highest for rituximab with a mean SI value of 5.2, followed natalizumab with 1.3, adalimumab with 0.9 and infliximab with 0.6 (Fig 4C).

### Evaluation of frequency of BP-specific CD4^+^ T cells in healthy donors

**Fig 5:**
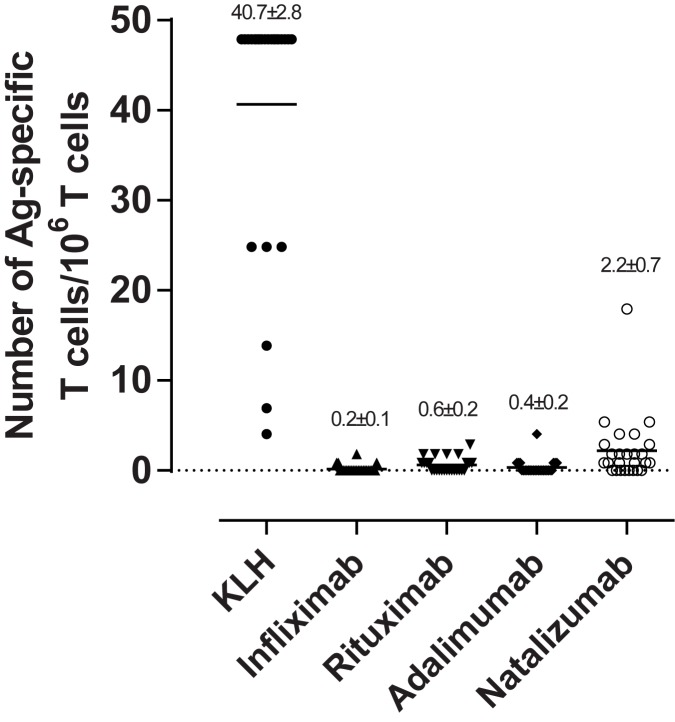
Frequencies of CD4^+^ T cells specific to the BPs. The frequency of CD4^+^ T cell specific for KLH, CMV, Infliximab, Rituximab, Adalimumab and Natalizumab expressed per 10^6^ naïve CD4^+^ T cell for the individual donors as well as the mean (± SEM) is indicated. The data is based on pooled data from proliferation at day six and eight from all 26 donors. The mean number of BP-specific CD4^+^ T cells identified is specified at the top of each histogram.

Based on proliferation at both day six and eight, we assessed the frequency of CD4^+^ T cells specific to the BPs (Fig 5). As described in the method sections, the calculations for determining the frequency of BP-specific CD4^+^ T cells is based on negative wells, and since all the donors in all wells responded positively to CMV, we could not estimate a frequency for this control. KLH demonstrated a strong T cell response, as the mean T cell frequency for KLH was 40.7 cells/10^6^ cells. The CD4^+^ T cell repertoire to the BPs was significantly lower, since the frequency of responding cells were 0.2 BP-specific CD4^+^ T cells/10^6^ cells specific to infliximab, 0.6 cells/10^6^ cells for rituximab, 0.4 cells/10^6^ cells for adalimumab and 2.2 cells/10^6^ cells for natalizumab.

### HLA-DR, DP and DQ haplotype frequencies

**Fig 6:**
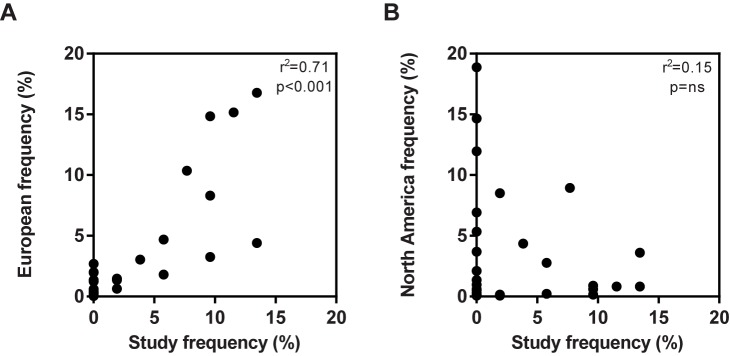
Frequency of donor HLA-DRB1. Comparison of the frequency of high resolution HLA-DRB1 allotypes expressed in the test population versus the; A) the European population and B) the North American population. The correlation was calculated using Pearson correlation coefficient.

The donors included in our study population were typed for the MHC class II alleles HLA-DR, -DP and -DQ to determine the representation of the population, see Table 2. The examination revealed that the study population covered all major HLA-DR, DP and DQ allotypes. To further assess the distribution of our analyzed donors, we compared the study population to the distribution of the European and North American population in terms of HLA-DRB1 (Fig 6). The donors showed multiple HLA-DRB1 allotypes, including the most frequent alleles present in the European population, where the correlation of frequencies in the populations was significant. This was not observed for the North American population.

**Table 2:** A summary of the high resolution HLA-DR, DQ and DP haplotypes of the included donors in the assay.

Donor	DRB1-1	DRB1-2	DRB3-1	DRB3-2	DRB4-1	DRB4-2	DRB5-1	DRB5-2	DQB1-1	DQB1-2	DQA1-1	DQA1-2	DPB1-1	DPB1-2	DPA1-1	DPA1-2
1	*13:02	*15:01	*03:01	-	-	-	*01:01	-	*06:02	*06:04	*03:01	*04:01	*01:01	*01:02	*01:03	-
2	*03:01	*04:04	*01:01	-	*01:01	-	-	-	*02:01	*03:02	*04:01	*04:02	*03:01	*05:01	*01:03	-
3	*12:01	*15:01	*02:02	-	-	-	*01:01	-	*03:01	*06:02	*04:02	*05:01	*01:01	*05:01	*01:03	*02:02
4	*11:01	*15:01	*02:02	-	-	-	*01:01	-	*03:01	*06:02	*04:02	*05:01	*01:01	*05:01	*01:03	-
5	*04:01	*04:04	-	-	*01:01	-	-	-	*03:02	-	*03:01	-	*03:01	-	*01:03	-
6	*13:02	*14:01	*02:02	*03:01	-	-	-	-	*05:03	*06:04	*04:01	-	*01:01	*01:02	*01:03	-
7	*13:01	*14:01	*01:01	*02:02	-	-	-	-	*05:03	*06:03	*04:01	*04:02	*01:01	*01:03	*01:03	-
8	*03:01	*04:04	*01:01	-	*01:01	-	-	-	*02:01	*03:02	*01:01	*03:01	*03:01	*05:01	*01:03	*02:01
9	*03:01	*07:01	*02:02	-	*01:03	-	-	-	*02:01	*03:03	*04:01	-	*02:01	*05:01	*01:03	-
10	*07:01	*13:01	*01:01	-	*01:01	-	-	-	*02:02	*06:03	*02:01	-	*01:03	*02:01	*01:03	-
11	*01:01	*13:02	*03:01	-	-	-	-	-	*05:01	*06:04	*06:01	*09:01	*01:01	*01:02	*01:03	*02:01
12	*04:04	*04:07	-	-	*01:01	-	-	-	*03:01	*03:02	*03:01	*19:01	*03:01	-	*01:03	*02:02
13	*07:01	*10:01	-	-	*01:01	-	-	-	*02:02	*05:01	*02:01	*11:01	*01:01	*02:01	*01:03	*02:01
14	*03:01	*13:02	*01:01	*03:01	-	-	-	-	*02:01	*06:04	*04:01	-	*01:02	*05:01	*01:03	-
15	*01:01	*07:01	-	-	*01:03	-	-	-	*03:03	*05:01	*04:01	*10:01	*01:01	*02:01	*01:03	*02:01
16	*03:01	*04:01	*01:01	-	*01:01	-	-	-	*02:01	*03:02	*04:01	-	*03:01	*05:01	*01:03	-
17	*15:01	-	-	-	-	-	*01:01	-	*06:02	-	*03:01	*15:01	*01:01	-	*01:03	*01:04
18	*11:01	*13:01	*01:01	*02:02	-	-	-	-	*03:01	*06:03	*02:01	*04:02	*01:03	*05:01	*01:03	-
19	*07:01	*15:01	-	-	*01:03	-	*01:01	-	*03:03	*06:02	*04:01	-	*01:02	*02:01	*01:03	-
20	*01:01	*01:02	-	-	-	-	-	-	*05:01	-	*02:01	*03:01	*01:01	-	*01:03	-
21	*01:01	*07:01	-	-	*01:01	-	-	-	*02:02	*05:01	*04:01	*11:01	*01:01	*02:01	*01:03	*02:01
22	*04:01	*04:04	-	-	*01:01	-	-	-	*03:02	-	*02:01	*04:02	*03:01	-	*01:03	-
23	*01:01	*15:01	-	-	-	-	*01:01	-	*05:01	*06:02	*02:01	*04:01	*01:01	*01:02	*01:03	-
24	*04:04	*15:01	-	-	*01:01	-	*01:01	-	*03:02	*06:02	*04:01	-	*01:02	*03:01	*01:03	-
25	*04:04	*14:01	*02:02	-	*01:01	-	-	-	*03:02	*05:03	*03:01	*04:01	*01:01	*03:01	*01:03	-
26	*04:01	*13:02	*03:01	-	*01:01	-	-	-	*03:01	*06:04	*02:01	*04:01	*01:02	*03:01	*01:03	-

### Responding donors

**Fig 7:**
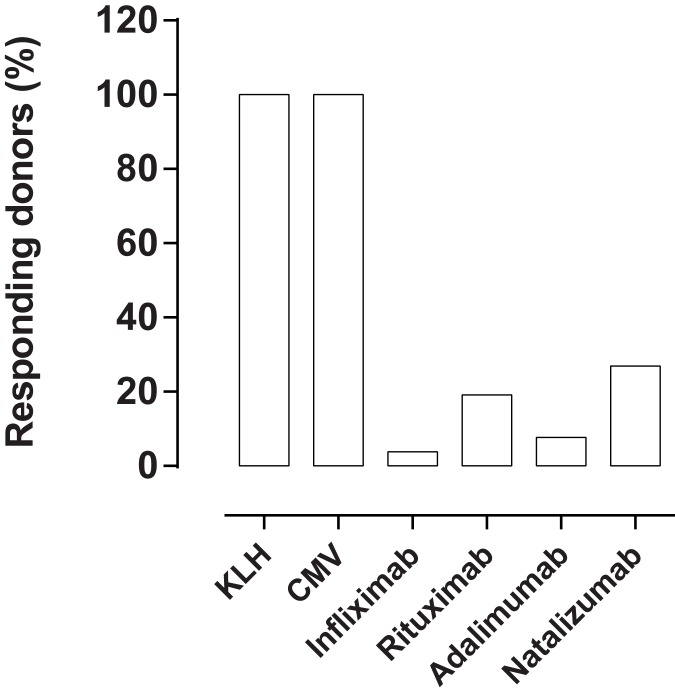
Frequency of responding donors. Summary of CD4^+^ T cell response to BPs among 26 donors. Donors were considered to be positive responders if one of both of the two proliferation assays, and/or the IL-2 secretion assay, showed a SI>2 with p<0.05 for a given donor’s response to a given BP compared to the respective control assay result.

**Fig 8:**
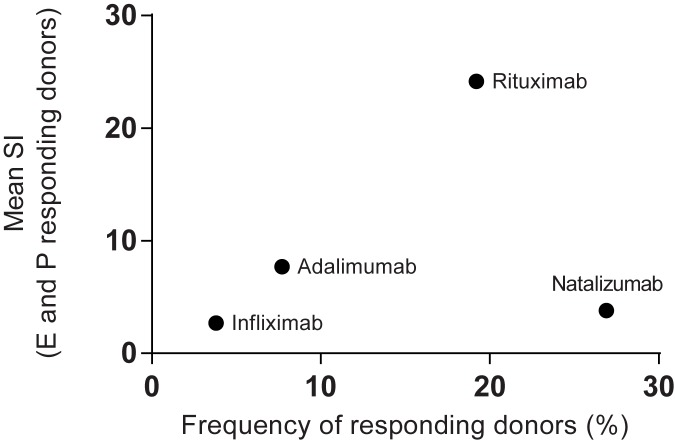
Plot of T cell response to the BPs. The frequency of responding donors and their mean IL-2 secretion and proliferation stimulation index (SI) levels. Donors were considered to be positive responders if one or both of the two proliferation assays, and/or the IL-2 secretion assay, showed an SI>2 with P<0.05 for a given donor’s response to a given BP compared to the respective control assay result. The mean SI for IL-2 secretion and proliferation levels for these positive responders are plotted against the % of positive responders for each BP.

The frequency of responding donors was calculated based on either proliferation or IL-2 secretion in the 26 donors (Fig 7). A similar approach has recently been demonstrated to correlate with the rate of clinical immunogenicity of biotherapeutic mAbs [43]. The naïve antigen KLH and the recall antigen CMV induced a response in all the donors analysed. Infliximab, rituximab, adalimumab and natalizumab, stimulated a response in 4%, 19%, 8% and 27% of the study population, respectively. To provide an assessment of the relative risk of each of the BPs to induce a T cell response, both the frequency of responders and the magnitude of the response was considered. Therefore, the percentage of responding donors was plotted against the mean SI of the responding donors in a heat plot (Fig 8). The BPs in the upper right region have a higher T cell response than the BPs in the lower left region. The results from this assay suggest that natalizumab and rituximab have a higher immunogenicity potential than adalimumab and infliximab. It shall be mentioned that one of the donors had a very high SI value for rituximab, which skewed the result in the plot.

## Discussion

Given the impact of immunogenicity on safety and efficacy of BPs, there is a rising interest in developing methods that clarify the immunogenic risks linked to therapeutic proteins [26]. For generation of high affinity ADAs, the process relies on T cell-dependent mechanisms. It has therefore been suggested that evaluating the presence of T cell epitopes by *in vitro* T cell assays in healthy donors can anticipate the risk of immunogenicity. Several considerations need to be accounted for when setting up such an assay. These include high sensitivity, high throughput and access to donor cohorts with HLA allotypes covering the most frequent HLA class II alleles in the major populations.

We chose to develop an assay that was based on CD4^+^-enriched T cells and irradiated PBMCs comprising the APC population, which is novel compared to the currently used CD8-depleted PBMC assay or DC:T cell assay. When using enriched CD4^+^ T cells instead of CD8^+^ T cell-depleted PBMCs, the amount of CD4^+^ T cells added to each well can be controlled, which is needed for T cell repertoire frequency calculations. Cytokine contribution from non-specific cells can also be limited. Using PBMCs as APCs eliminates the time used to generate DCs hence allowing high throughput. To quantify the T cell response, two independent readouts for T cell activation were exploited; T cell proliferation measured by ^3^[H]-thymidine incorporation and IL-2 secretion detected by ELISpot. A major challenge for *in vitro* immunogenicity prediction assays for BPs is the weak response. A high sensitivity is needed since frequencies of naïve T cells specific for a foreign antigen are in the range of 0.3–70 cells/10^6^ [17, 44, 45]. Few donors responded to the BPs when assessing proliferation by thymidine incorporation. The limitation with this method is that it only conveys a snapshot of what happens, and consequently events occurring at an earlier or later time-point can be missed. An alternative to increase sensitivity could be a flow cytometry-based approach using CFSE labelling, Ki67 expression or EdU incorporation [46]. In contrast, ELISpot is a highly sensitive method that allows for detection of a single cell that secrets a protein [47, 48]. Most positive responses were thus captured with this method. Other contributing factors to the increased sensitivity of the ELISpot assay could be the addition of the co-stimulatory anti-CD28/CD49d Abs and the re-challenge with BPs, which were not applied in the proliferation assay. To distinguish responding donors from non-responding donors we used a combination of two criteria, SI and statistical difference. A SI value above 2 was defined a positive response, which is equivalent to other similar studies that have defined their SI cut-off value ranging from 1.8 to 3 [16, 49, 50]. The SI value was pre-set to be above 2 to achieve minimum signal-to-noise, maximum sensitivity and limit false positive events.

To evaluate the performance of the T cell:PBMC assay, we assessed 26 healthy, drug-naïve donors for their T cell response to KLH, CMV and the BPs. Use of healthy drug-naïve donors are current practice in the field of Ab analysis, as they better reflect the patient population compared with BP-treated patients that already have developed ADAs. Moreover, as most of the BPs are used for treatment of several diseases, each with different co-medication that can affect the ADA response, we wanted to evaluate the assay independent of disease indication, which again could have impact on the correlation between T cell assay and ADA development [50]. The haplotype of the donors included in the study were determined retrospectively and was found to have a frequency of HLA-DRB1 types that correlated to the European population. HLA class II molecules determine the sequence of the peptides that can be bound and presented to the T cells, and it is therefore essential to identify the population for which the HLA profile is relevant [7]. Since the current study population represents the European population, the results were subsequently compared to ADA incidences reported in Europe, as noted in Table 1.

One of the approaches to evaluate the T cell response was to calculate the number of BP-specific T cells. The magnitude of the T cell response to mAbs has recently been demonstrated to depend on the number of pre-existing antigen-specific T cells [19]. In the current study, the KLH-specific T cell repertoire was calculated to be 41 cells/10^6^ cells, which is similar to other studies that have reported the KLH-specific T cell frequency to be 19–42 [51], 5–30 [19] and 10–70 cells per 10^6^ CD4^+^ T cells [17]. The frequency of BP-specific CD4^+^ T cells was found to be in the range of 0.2–2.2 cells/10^6^ CD4^+^ T cells, which correlates well with the findings of Delluc and colleagues [19]. They observed a frequency of CD4^+^ T cells to infliximab, rituximab and adalimumab to be 0.2, 0.4 and 0.3 cells/10^6^ T cells, respectively. For the mAbs, the T cell responses are largely mediated by naïve T cells [19] due to the foreignness of the complementarity determining regions (CDRs) and for some donors the frame work as well, but it could also be caused by memory cells as many patients have pre-existing Abs, including rheumatoid factors, anti-allotype, anti-hinge and anti-glycan Abs [52].

A direct comparison of *in vitro* T cell responses to clinical data cannot be conducted as many factors complicate the determination of the “true” immunogenicity of a BP, including differences in clinical trial testing, time-frame over which the ADA response is measured, use of different ADA assays and differences in reporting. An example is rituximab treatment that is well accepted in cancer patients [53, 54], but not in systemic lupus erythematosus patients, since in the latter ADA responses are found in up to 36% of the patients [36]. In the current assay, rituximab was observed to stimulate a response in 19% of the donors analyzed. Considering both the frequency and the magnitude of the response, the assay predicts rituximab to be immunogenic, which correlates well with the overall clinical picture (Table 1). For infliximab and adalimumab on the other hand, surprisingly low frequencies of responding donors were found; 4% and 8%, respectively. The occurrence of ADAs in the clinic to infliximab and adalimumab is generally high, ranging from 4–87% for patients with autoimmune diseases. Moreover, based on the degree of foreignness, infliximab can be expected to induce a higher T cell response than adalimumab since adalimumab is fully human and infliximab is chimeric and therefore has more non-human sequences. However, an important factor is the mode of action of the BP in question. Many BPs can modulate the T cell response directly or affect the APCs, hence potentially influence Ag-uptake, presentation and cytokine profile [55, 56]. Anti-TNF-α mAbs have been shown to affect the maturation and survival of APCs, as well as suppress T cell proliferation [57, 58]. Therefore, TNF-α inhibitors are likely to interfere with the assay, resulting in suppressed proliferation and consequently an underestimated response. Two recent papers have assessed the *in vitro* T cell response to rituximab, infliximab and adalimumab using either the EpiScreen^TM^ DC:T cell assay or the EpiScreen^TM^ Time Course T cell (PBMC) assay at Antitope Ltd. [43, 50]. Karle *et al*. observed that the mAbs had the ability to initiate a T cell response in 10, 14 and 20% of the donors for rituximab, adalimumab and infliximab, respectively [50], whereas Joubert *et al*. observed rituximab, adalimumab and infliximab to induce a response in 10, 21 and 14% of the analyzed donors [43]. These discrepancies underline the difficulties in assessing the immunogenicity potential of Abs binding to targets affecting immune cells. Furthermore, natalizumab was found to induce a response in 27% of the donors. Natalizumab is in general believed to be a weak immunogen with detected Abs in 4–5% in phase I and II studies with patients with multiple sclerosis. [42]. However, these numbers are likely underestimated as the long-term immunogenicity of natalizumab is unknown. Indeed, recent studies conducted within the ABIRISK project show that natalizumab generates ADAs in a much higher frequency than previously reported (unpublished data by ABIRISK).

Due to the abovementioned factors, it is difficult to predict the clinical immunogenicity with an *in vitro* T cell assay. However, it gives the possibility to assess whether the compounds have the capacity to induce a T cell response. The test compounds should be viewed individually and not ranked against each other. Even though the target is the same, as for infliximab and adalimumab, formulation and injection routes are different, which likely also influence the clinical immunogenicity. Rather, *in vitro* T cell assays can be used to support lead candidate selection during drug development by choosing a variant with a low T cell response.

In conclusion, we have developed a novel T cell:PBMC assay that can evaluate the immunogenicity potential of BPs. It has the capability of detecting low frequencies of BP-specific T cells, and demonstrates a high *in vitro* immunogenicity to several BPs with a documented high clinical immunogenicity. The assay provides information that in conjugation with other immunogenicity prediction tools can be used in early drug development to select drug candidates with low immunogenicity potential to ultimately increase patient safety.

## Supporting information

S1 File Written information regarding granted permission to publish Fig 1. (DOCX)
