# Supplementary material for: Quantitative analysis of the CD4+ T cell response to therapeutic antibodies in healthy donors using a novel T cell:PBMC assay
Source: PLoS One. 2017 May 31;12(5):e0178544. doi: 10.1371/journal.pone.0178544 (PMC5451071; doi:10.1371/journal.pone.0178544)
Supplement: S1 File — (DOCX) [file pone.0178544.s001.docx]

**Supporting information file**

Written information regarding granted permission to publish figure 1:

Dear Heidi,

Thanks for your message. You are free to use Servier Medical Art images as you want. As stated in <http://servier.com/Powerpoint-image-bank> :

“Servier Medical Art by [Servier](http://www.servier.com/Powerpoint-image-bank) is licensed under a [Creative Commons Attribution 3.0 Unported License](http://creativecommons.org/licenses/by/3.0/)

Please do not forget to cite Servier Medical Art in your references.

Best regards,

Xavier GALLET

Servier International
